# Anti-Inflammatory Effects of *Artemisia argyi* H. Fermented by *Lactobacillus plantarum* in the LPS-Induced RAW 264.7 Cells and DSS-Induced Colitis Model

**DOI:** 10.3390/foods13070998

**Published:** 2024-03-25

**Authors:** Ji Yun Lee, Ji-Hyun Kim, Ji Myung Choi, Byeong Wook Noh, Hyun Young Kim, Eun Ju Cho

**Affiliations:** 1 Department of Food Science and Nutrition, Pusan National University, Busan 46241, Republic of Korea; wldbslee1246@pusan.ac.kr (J.Y.L.); llissunll@gmail.com (J.-H.K.); qud7841@pusan.ac.kr (B.W.N.); 2 Department of Food and Nutrition, Kyungsung University, Busan 48434, Republic of Korea; poutia@naver.com; 3 Department of Food Science and Nutrition, Gyeongsang National University, Jinju 52725, Republic of Korea

**Keywords:** *Artemisia argyi* H., ulcerative colitis, dextran sulfate sodium, RAW 264.7 cells, anti-inflammation

## Abstract

Ulcerative colitis is a chronic inflammatory disease caused by abnormal immune responses in the intestinal mucosa and gut microorganisms. Unlike other mugworts, *Artemisia argyi* H. (*A. argyi* H.) enhances antioxidant, anti-inflammatory, and anticancer effects, but the improvement effects against gut inflammation have not yet been reported. Therefore, this study aimed to confirm the alleviation of the inflammatory state in the gut by *A. argyi* H. fermented with *Lactobacillus plantarum* (FAA), using lipopolysaccharide (LPS)-induced RAW 264.7 cells and dextran sulfate sodium (DSS)-induced colitis models. In vitro, FAA (10, 50, 100, and 200 μg/mL) was pretreated into RAW 264.7 cells, followed with LPS (100 ng/mL), which induced the cell damage. Meanwhile, in vivo, FAA (100, 200 mg/kg/day) was orally administered into 6-week-old C57BL/6N mice for 3 weeks. During the last week of FAA administration, 2.5% DSS was used to induce colitis. The results showed that FAA reduced the production of nitric oxide (*p* < 0.0001), tumor necrosis factor (TNF)-α, interleukin (IL)-6 (*p* < 0.0001), and IL-1β (*p* < 0.0001) in the LPS-induced RAW 264.7 cells. Moreover, in the DSS-induced colitis model, FAA alleviated clinical symptoms (*p* < 0.001), inhibited the inflammatory state by reducing the production of TNF-α (*p* < 0.0001) and interferon-γ in intestinal immune cells (*p* < 0.0001), and strengthened the intestinal barrier by increasing the number of goblet cells (*p* < 0.0001). Furthermore, the anti-inflammatory effects were confirmed by the alleviation of histological damage (*p* < 0.001) and down-regulation of the expression of inflammatory proteins (TLR4, *p* < 0.0001; MyD88, *p* < 0.0001; Cox-2, *p* < 0.0001). These results suggest the potential of FAA as a dietary ingredient for preventing inflammation in the gut.

## 1. Introduction

Inflammatory bowel disease (IBD) is a chronic inflammatory disease characterized by an abnormal immune response in the gastrointestinal tract and is largely divided into ulcerative colitis and Crohn’s disease [1]. IBD can be caused by an abnormal immune response of the intestinal mucosa, the state of the gut microorganisms, damage to the intestinal mucosal barrier, and genetic and environmental factors [2]. However, its exact etiology and pathophysiology have yet to be revealed, and it is becoming a problem worldwide [3].

Unlike Crohn’s disease, which mainly occurs in the small intestine, ulcerative colitis is characterized by occurring in the rectum and colon [4]. However, generally, colitis is known to be caused by age, sex, race, ethnicity, genetics, smoking, diet, gut microbiota, and appendectomy [5]. The drugs used to treat colitis include anti-inflammatory drugs such as corticosteroids, 5-aminosalicylic acid, and sulfasalazine (SASP) [6]. However, these drugs have serious side effects such as nausea, headache, and skin rashes; furthermore, many studies have attempted to find the possibility of using natural materials with low toxicity and no side effects for the prevention and improvement of colitis [7].

Macrophages play important roles in the immune system [8]. They differentiate from monocytes and engulf and digest cellular debris, pathogens, and other foreign material [9]. In a colitis model, an increase in macrophages and abnormal activation of inflammatory colon tissues were observed [10]. Activated macrophages secrete pro-inflammatory cytokines and inflammatory proteins that cause intestinal damage [11]. Ethanol extracts of *Artemisia gmelinii* and *Artemisia argyi* prevent inflammatory symptoms in lipopolysaccharide (LPS)-induced RAW 264.7 cells, alleviate colitis symptoms, and improve immune regulatory functions [12,13]. Additionally, soymilk fermented with *Lactococcus lactis* subsp. prevents inflammation in LPS-induced RAW 264.7 cells and is known to be effective in alleviating inflammatory symptoms in colitis models [14].

Moreover, the composition of the gut microbial community or an imbalance in gut microorganisms has an important correlation with colitis models. In other words, the number of beneficial bacteria changes, and potentially harmful bacteria may over-proliferate in the colitis model [15]. Additionally, an imbalance in the gut microbiota can cause an abnormal immune response, which can mistakenly attack the intestinal lining, exacerbate inflammation, and contribute to the development or progression of colitis [16]. Regulation of the gut microbial community serves as a key factor in the prevention and treatment of colitis [17].

Artemisia species have been used in traditional medicine to treat digestive disorders, typhoid, epilepsy, and kidney disease [18]. *Artemisia argyi* H. (*A. argyi* H.) belongs to the Artemisia species; it is cultivated in the Namhae region of Gyeongsangnam-do and is a domestic unique resource registered as a variety protection material by the Korea Forest Service in 2013 [19]. *A. argyi* H. belongs to the medicinal mugwort family and contains more active substances, such as jaceosidin and eupatilin, than other artemisia species [20]. Meanwhile, fermentation of the plant produces probiotics and helps to increase the amount of phytochemicals [21]. Therefore, the fermented plant shows high nutritional value and has significant biological properties, such as antioxidant, anti-inflammation, and immunomodulatory activities, compared to the non-fermented plant [22]. Our previous study reported that the fermented *A. argyi* H. with *L. plantarum* (FAA) contained a more bioactive component, jaceosidin, than non-fermented *A. argyi* H. with *L. plantarum* [23]. Furthermore, the immune enhancement effect and antioxidant activity of FAA were demonstrated [24]. These findings suggested its potential as a functional material for preventing and improving inflammatory responses. Based on these evidences, the present study hypothesized that FAA may play a key role as immune regulator and thus investigated its anti-inflammatory effects using both LPS-induced RAW 264.7 cells and a dextran sulfate sodium (DSS)-induced colitis mouse model.

## 2. Materials and Methods

### 2.1. Reagents

Dulbecco’s Modified Eagle’s Medium (DMEM), roswell park memorial institute (RPMI)-1640, fetal bovine serum (FBS), and penicillin-streptomycin were purchased from Welgene Inc. (Daegu, Korea). Griess reagent, lipopolysaccharides from Escherichia coli O111:B4, and concanavalin A from Canavalia ensiformis (Jack bean) were obtained from Sigma Chemical Co. (Saint Louis, MO, USA). MTS CellTiter 96 Aqueous One Solution Assay was purchased from Promega Co., (Madison, WI, USA). Interleukin (IL)-6, Mouse tumor necrosis factor (TNF)-α, and Interferon-gamma (IFN-γ) enzyme-linked immunosorbent assay (ELISA) kit were bought from R&D systems (Minneapolis, MN, USA). Mouse IL-1 beta (β) ELISA kit was obtained from Abcam (Cambridge, MA, USA). PowerFecal Pro DNA kit was purchased from QIAGEN (Hilden, Germany). All primers (Table 1) for investigating the bacterial composition were obtained from Macrogen (Seoul, Republic of Korea). Radioimmunoprecipitation assay (RIPA) buffer was from Elpis Biotech. (Daejeon, Republic of Korea). Protease inhibitor cocktail was bought from Calbiochem (Cambridge, MA, USA). 2-mercaptoethanol was obtained from Biopure (Cambridge, MA, USA). 2x Laemmli sample buffer and enhanced chemiluminescence (ECL) substrate solution were purchased from Bio-rad Laboratories (Hercules, CA, USA). Polyvinylidene fluoride (PVDF) membranes were supplied from Millipore (Billerica, MA, USA). Primary and secondary antibodies were obtained from Cell Signaling Tech. (Beverly, MA, USA) and Santa Cruz Biotech (Dallas, TX, USA).

### 2.2. Sample Preparation

*A. argyi* H. was supplied from the farming association of *A. argyi* H. in Namhae [23], and FAA was processed by the following procedures [24]. The *A. argyi* H. leaves were steamed at 100 °C for 5 min, then crushed in malt extract broth. Afterward, these extracts were sterilized, and it was used as a fermentation substrate. MRS agar medium inoculated with *Lactobacillus plantarum* was shaken at 150 rpm for 2 days at 30 °C. Then, it was mixed with the *A. argyi* H. at a 5% volume ratio and fermented at 30 °C for 4 days to produce FAA. FAA was freeze-dried and filtered using ethanol as an extraction solvent, and the filtrate was concentrated under reduced pressure using a rotary vacuum evaporator.

### 2.3. Cell Culture

The RAW 264.7 mouse macrophage cell line, was obtained from Korea cell line bank (KCLB, Seoul, Republic of Korea). The composition of cell culture medium was DMEM containing 10% FBS and 1% penicillin-streptomycin [25]. RAW 264.7 cells were cultured in the complete medium at 37 °C in a humidified 5% CO_2_ incubator.

### 2.4. Measurement of Nitric Oxide (NO)

RAW 264.7 cells were seeded in a 96-well plate at a density of 2 × 10^4^ cells/well and incubated for 24 h [26]. The ethanol extract of FAA was pretreated at different concentrations (10, 50, 100, and 200 μg/mL) for 2 h, followed with LPS at 100 ng/mL, and incubated for 24 h. On the third day, the cell supernatant was collected, and a mixture (1:1) of supernatant and Griess reagent were incubated for 15 min blocking light. The absorbance was measured at 540 nm and calculated as a percentage of 100% of cells from the normal group.

### 2.5. Measurement of Cytokines Production

To evaluate TNF-α, IL-6, and IL-1β, RAW 264.7 cells were seeded at a density of 2 × 10^4^ cells/well in a 96-well plate and incubated for 24 h [27]. FAA was pretreated for 2 h, followed with LPS at 100 ng/mL, and incubated for 24 h. After incubation for 24 h, the cell supernatant was collected and used for measuring cytokine production by ELISA kits, according to the manufacturer’s instructions.

### 2.6. Animals and Experimental Designs

**Figure 1:**
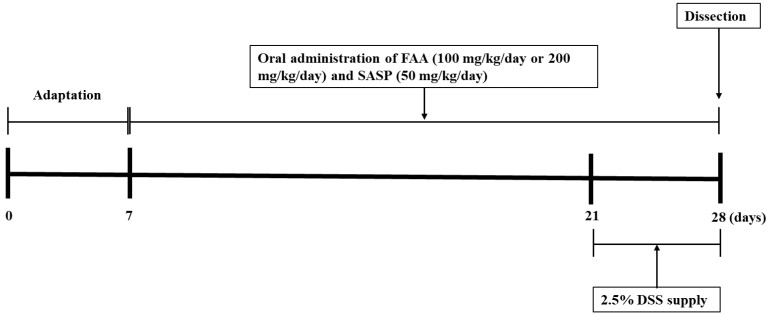
Experimental schedule.

Six-week-old male C57BL/6N mice (17–21 g) were purchased from Orient Bio Inc. (Seongnam, Republic of Korea). The mice were raised in a laboratory animal room and freely drank and were fed a normal diet. Moreover, the laboratory environment was controlled with temperature (20 ± 2 °C) and humidity (55 ± 5%) in a 12-h light/dark cycle, with a supply of water. After a one-week adaptive period, the mice were randomly grouped into five groups, with nine mice in each group (*n* = 9). The mice were orally administered the ethanol extract of FAA at different concentrations or SASP for 3 weeks, except those in the normal group [28]. To establish a DSS-induced colitis model, after two weeks of treatment, the groups treated with the experimental samples, except for the normal group, were supplied with 2.5% DSS in their drinking water (Figure 1). Consequently, this study planned to be composed of the following five treatment groups: Normal (pure water supply + oral administration of pure water), Control (2.5% DSS supply + oral administration of pure water), FAA 100 (2.5% DSS supply + oral administration of FAA in dose of 100 mg/mL), FAA 200 (2.5% DSS supply + oral administration of FAA in dose of 200 mg/mL), and SASP 50 (2.5% DSS supply + oral administration of SASP in dose of 50 mg/mL). The mice body weights were evaluated once a week, and samples for oral administration were prepared every 3 days. Additionally, a behavioral test was implemented 3 days before dissection for 2 days. On the day of dissection, the mice were deeply anesthetized using an anesthetic containing ZoletilTM, Rompun^®^, and 0.9% NaCl, and subsequently dissected. To evaluate hepatotoxicity, tissue samples were collected from the colon, small intestine, and brain, and blood samples were collected from the heart. All experimental procedures involving mice were approved by the Animal Care and Use Committee of Pusan National University (PNU-2022-0048).

### 2.7. Measurement of Colon Length and Disease Activity Index (DAI)

Colon length was measured immediately after sample collection. The DAI was used to measure the inflammation criteria [29], which consisted of rectal bleeding, stool consistency, and weight loss (percentage of initial weight) (Table 2). Scores were obtained using clinical parameters during the treatment period. 

### 2.8. Isolation of Mesenteric Lymph Node (MLN)

The MLN cells were isolated by collecting them and washing them in PBS with 5% FBS and 2% penicillin/streptomycin. Subsequently, they were separated using a 70 μm cell strainer. The MLN cells were rinsed with RPMI-1640 medium containing 5% FBS and 2% penicillin/streptomycin (1500 rpm, 10 min). Then, red blood cells were removed using ACK lysis buffer. Finally, the MLN cells were rinsed with RPMI-1640 medium and were suspended in RPMI-1640 medium containing 10% FBS and 1% penicillin/streptomycin [30].

### 2.9. Measurement of Cytokine Production

TNF-α and IFN-γ were determined using an enzyme-linked immunosorbent assay (ELISA) kit. The MLN cells were seeded in a U-bottomed 96-well plate at a density of 1 × 10^5^ cells/well, and 5 μg/mL of ConA was added to the plate [31]. Subsequently, the plates were incubated for 48 h (37 °C, 5% CO_2_). After centrifugation at 300× *g* for 10 min, the cytokines in the supernatant were quantitated by measuring the absorbance at 450 nm.

### 2.10. Histological Analysis

Colonic sections (1 cm thick) from the end of the cecum were fixed in 4% formaldehyde. The sections were subsequently embedded in paraffin and stained with hematoxylin and eosin (H&E) and periodic acid-Schiff (PAS). The degree of colitis was scored using the criteria presented in Table 3. Briefly, colon sections were graded according to the severity and extent of inflammation, and crypt damage [32]. Each value was multiplied by an extent index reflecting the extent of the involvement range (1: 0–25%, 2: 26–50%, 3: 51–75%, 4: 76–100%). Three sections of each colon were evaluated for each score. To measure the number of goblet cells by PAS staining, the percentage of positively stained cells in the area of interest (AOI) was calculated using ImageJ software (Version 1.53k) [33].

### 2.11. Fecal Collection and DNA Extraction

Before the dissection, the feces were collected within 30 min and were stored at −80 °C temperatures until the DNA was extracted. Bacterial DNA was isolated from the feces (250 mg each) using a DNA extraction kit according to the manufacturer’s instructions.

### 2.12. Quantitative Real-Time PCR (qPCR)

Genomic DNA was extracted using PowerFecal Pro DNA kit. DNA was evaluated by 16S rRNA sequencing, and the purity of the samples was measured using an iSeq platform (Illumina, San Diego, CA, USA). Identity of the gut microbiota was quantified at the absorbance ratios at 260/280 nm and amplified using qPCR (CFX Connect Real-Time PCR Detection System, Bio-Rad (Hercules, CA, USA)). PCR conditions were 95 °C for 5 min and 40 cycles at 95 °C for 5 s at the annealing temperature. The primer specificity was verified as follows: 5 min at 65 °C, 5 min at 95 °C, and 0.5 °C increments [34].

### 2.13. Western Blot Analysis

The colon tissues were collected and homogenized with lysis buffer, which consisted of RIPA buffer with protease inhibitor cocktail. Then, the homogenized samples were centrifuged at 4 °C and 12,000 rpm for 30 min, repeating twice. After centrifuging, the supernatant was quantified by Bradford assays. The Western samples used for analysis were prepared by mixing the cell supernatant from which proteins had been quantified with sample buffer, which consisted of 2-mercaptoethanol and 2x Laemmli sample buffer (1:1, *v*/*v*). The samples were loaded and subjected to electrophoresis for 2 h at 90 V on 8–13% sodium dodecyl sulfate polyacrylamide gels (SDS-PAGE) and then transferred to the PVDF membrane for 120 min at 90 V. Then, the membrane was blocked for 1 h with 5% skim milk or BSA in PBS-T, washed three times for 10 min each, and incubated with the primary antibodies at 4 °C overnight. The primary antibodies used in this study were β-actin (1:1000; Cell Signaling Tech. Beverly, MA, USA); cyclooxygenase-2 (COX-2, 1:500; Cell Signaling Tech. Beverly, MA, USA); toll-like receptor 4 (TLR4, 1:200; Santa Cruz Biotech., Dallas, TX, USA); and myeloid differentiation primary response 88 (MyD88, 1:1000; Cell Signaling Tech. Beverly, MA, USA). After overnight incubation, the secondary antibody was attached at room temperature for 1 h, and targeted protein expression was measured with the ECL solution by using the Davinci-chemiluminescent imaging system (CoreBio, Seoul, Republic of Korea).

### 2.14. Statistical Analysis

The data were represented as mean ± standard deviation (SD). Statistics were analyzed using the SPSS program (version 26.0). One-way analysis of variance (ANOVA) by Tukey’s multiple range test was performed. The significance level was shown as * *p* < 0.05, ** *p* < 0.005, *** *p* < 0.001, **** *p* < 0.0001 for all statistics.

## 3. Results

### 3.1. Effects of FAA on NO Production in the LPS-Induced RAW 264.7 Cells

**Figure 2:**
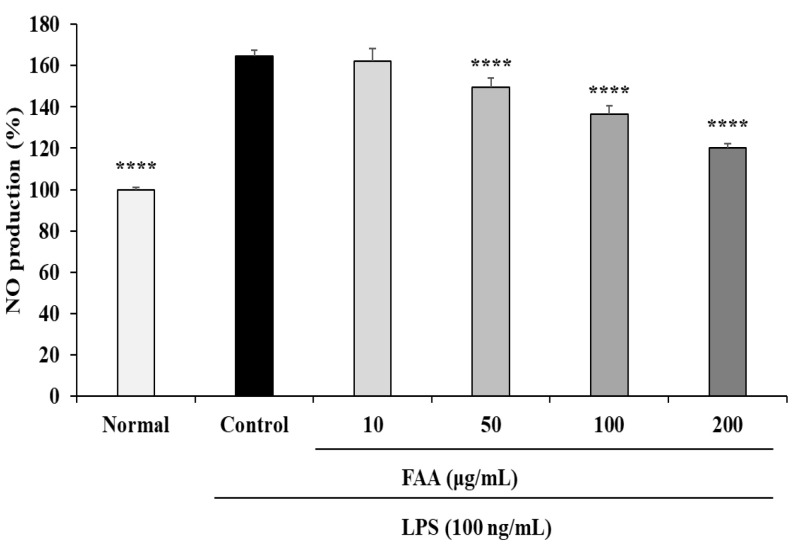
Effects of fermented *A. argyi* H. (FAA) on NO production in LPS-induced RAW 264.7 cells. Values are mean ± SD. **** *p* < 0.001 vs. Control by one-way ANOVA and Tukey’s multiple comparison test. ‘Normal’ group represents the non-treated cells, ‘Control’ group represents the LPS-treated cells, ‘10’, ‘50’, ‘100’, and ‘200’ groups represent the four concentrations of FAA treatment (10, 50, 100, 200 μg/mL) in LPS-treated cells.

When the RAW 264.7 cells were treated with the ethanol extract of FAA through different concentrations (1, 5, 10, 25, 50, 100, and 200 μg/mL), the cell viability was significantly increased in a dose-dependent manner, compared to the normal cells. Among these concentrations of FAA, the four concentrations with high viability (10, 50, 100, and 200 μg/mL) were selected. The RAW 264.7 cells were exposed to 100 ng/mL LPS to investigate inflammatory responses through NO secretion. LPS markedly induced NO secretion compared to the normal group (164.76%) (Figure 2). However, the pre-treatment groups of FAA (50, 100, and 200 μg/mL) significantly inhibited the production of NO in a dose-dependent manner, to 149.61%, 136.47%, and 120.14%, respectively.

### 3.2. Effects of FAA on Cytokine Production in the LPS-Induced RAW 264.7 Cells

**Figure 3:**
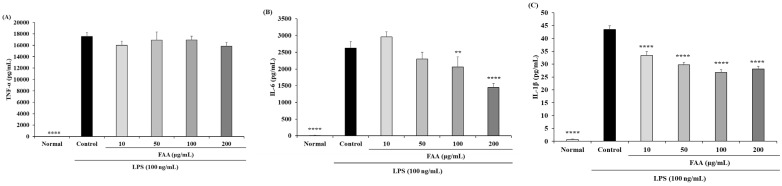
Effects of fermented *A. argyi* H. (FAA) on cytokine production in the LPS-induced RAW 264.7 cells. (**A**) TNF-α, (**B**) IL-6, and (**C**) IL-1β. Values are mean ± SD. ** *p* < 0.01, **** *p* < 0.0001 vs. Control by one-way ANOVA and Tukey’s multiple comparison test. ‘Normal’ group represents the non-treated cells, ‘Control’ group represents the LPS-treated cells, ‘10’, ‘50’, ‘100’, and ‘200’ groups represent the four concentrations of FAA treatment (10, 50, 100, 200 μg/mL) in LPS-treated cells.

The supernatant was used to investigate the production of pro-inflammatory cytokines; the supernatant of the RAW 264.7 cells was used. LPS induced prominently increased amounts of TNF-α, IL-6, and IL-1β compared to each of the normal groups, showing 15,850.24 pg/mL, 2627.23 pg/mL, and 43.48 pg/mL, respectively (Figure 3). The production of IL-6 in the pre-treatment of FAA was significantly reduced in the 50, 100, and 200 μg/mL groups, to 2299.20 pg/mL, 2060.74 pg/mL, and 1449.07 pg/mL, respectively (Figure 3B). The secretion of IL-1β was significantly inhibited by all concentrations of FAA, to 33.35 pg/mL, 29.73 pg/mL, 26.74 pg/mL, and 28.13 pg/mL, respectively (Figure 3C). These results indicate that the production of pro-inflammatory cytokines was significantly increased by induction with LPS, while the administration of FAA inhibited the production of cytokines.

### 3.3. Effects of FAA on Colon Length in the DSS-Induced Colitis Model

**Figure 4:**
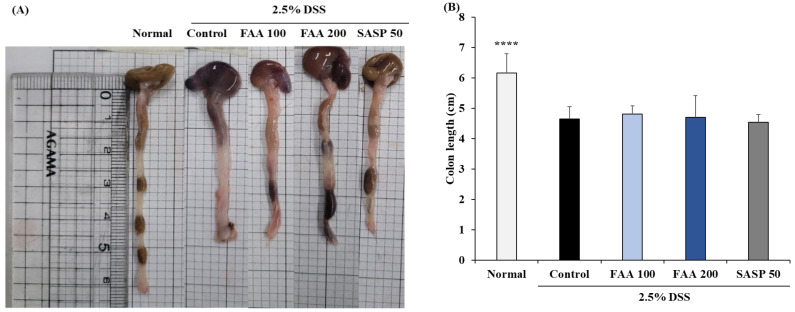
Effects of fermented *A. argyi* H. (FAA) on representative photos (**A**) and colon length (**B**) in the DSS-induced colitis model. Values are mean ± SD (*n* = 9). **** *p* < 0.001 vs. Control by one-way ANOVA and Tukey’s multiple comparison test. Normal (pure water supply + oral administration of pure water), Control (2.5% DSS supply + oral administration of pure water), FAA 100 (2.5% DSS supply + oral administration of FAA in dose of 100 mg/mL), FAA 200 (2.5% DSS supply + oral administration of FAA in dose of 200 mg/mL), and SASP 50 (2.5% DSS supply + oral administration of SASP in dose of 50 mg/mL).

The colon length was shortened in all DSS-treated groups by 4.64 cm, compared to that in the normal group, verifying the progression of colitis by DSS (Figure 4A,B). The colon lengths in the FAA 100 and FAA 200 groups were 4.81 and 4.7 cm, respectively, but none of the FAA and SASP 50 groups showed a significant difference in colon length.

### 3.4. Effects of FAA on DAI in the DSS-Induced Colitis Model

**Figure 5:**
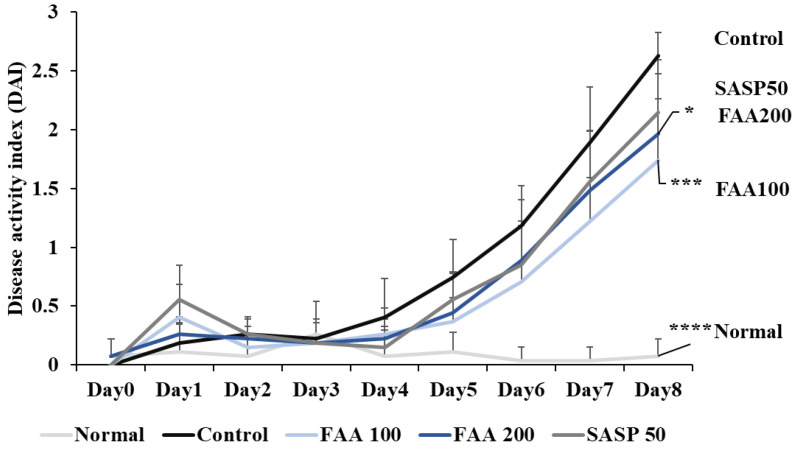
Effects of fermented *A. argyi* H. (FAA) on DAI in the DSS-induced colitis model. Values are mean ± SD (*n* = 9). * *p* < 0.05, *** *p* < 0.001, **** *p* < 0.001 vs. Control by one-way ANOVA and Tukey’s multiple comparison test. The mice were grouped and treated as described in Figure 4.

From the 5th day, the DAI score of the control group started increasing, compared with those of the other groups (Figure 5). On the 8th day, the DAI score of the control group was the highest, and the FAA and SASP 50 groups showed attenuated DAI scores, which were increased by DSS. These results demonstrated that FAA effectively relieved the symptoms of colitis.

### 3.5. Effects of FAA on the Production of Cytokines in the DSS-Induced Colitis Model

**Figure 6:**
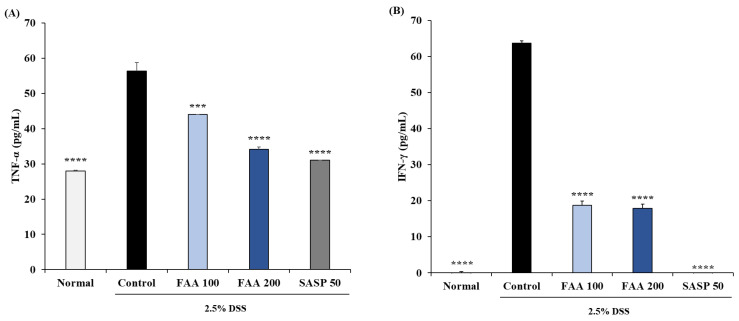
Effects of fermented *A. argyi* H. (FAA) on the production of cytokines in the DSS-induced colitis model. (**A**) TNF-α and (**B**) IFN-γ were treated by ConA for 24 h. Cytokine production was determined by ELISA kit. Values are mean ± SD (*n* = 9). *** *p* < 0.001, **** *p* < 0.001 vs. Control by one-way ANOVA and Tukey’s multiple comparison test. The mice were grouped and treated as described in Figure 4.

To evaluate the attenuation of inflammatory responses, secretion of pro-inflammatory cytokines was observed in MLN cells. TNF-α is secreted in the acute stage of colitis, and IFN-γ is secreted in the chronic stage of colitis. The production of TNF-α in the control group was higher than in the normal group (56.31 pg/mL), and FAA 100 and FAA 200 relieved the production of TNF-α compared to the control group, showing 43.98 pg/mL and 34.17 pg/mL (Figure 6A). The secretion of INF-γ in the only DSS-treated group showed a prominent increase (63.73 pg/mL), compared to the normal group, and FAA 100 and FAA 200 also attenuated the concentration of IFN-γ, compared to the control group, to 18.72 pg/mL and 17.86 pg/mL, respectively (Figure 6B). Although FAA did not affect T-cell proliferation, these results demonstrated that FAA has anti-inflammatory effects by inhibiting the production of Th-type cytokines, which are TNF-α and INF-γ in MLN cells.

### 3.6. Effects of FAA on Histopathology in the DSS-Induced Colitis Model

**Figure 7:**
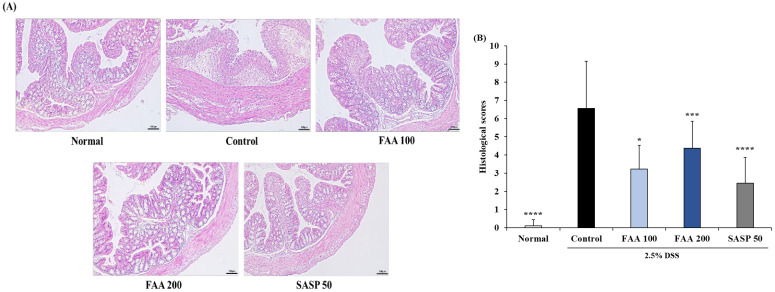
Effects of fermented *A. argyi* H. (FAA) on histological analysis for inflammation extent in the DSS-induced colitis model. (**A**) The colon section was stained with H&E. Original magnification: 10× (bar = 100 μm). (**B**) The histopathological scores were analyzed by the criteria. Values are mean ± SD (*n* = 9). * *p* < 0.05, *** *p* < 0.001, **** *p* < 0.001 vs. Control by one-way ANOVA and Tukey’s multiple comparison test. The mice were grouped and treated as described in Figure 4.

To detect the amelioration of histopathologic symptoms with FAA, it was evaluated by the presence of edema, degree of injury, infiltration of leukocytes, ulceration, and destruction of crypts through histology of the colon sections using H&E staining (Figure 7). The colon sections of the control group exhibited inflammatory changes in colonic architecture, crypt damage, and mixed-cell infiltration composed of macrophages and leukocytes, compared to the normal group. However, histological analysis of the colon for FAA exhibited reduced cell infiltration and a reduced degree of mucosal edema and injury (Figure 7A). Through the assessment of the intestinal inflammatory status, the control group exhibited significantly increased histopathological scores compared to the normal group (Figure 7B). However, the FAA 100 and FAA 200 groups exhibited a significant decrease compared to the control group. These findings suggest that FAA may relieve the symptoms of colitis.

**Figure 8:**
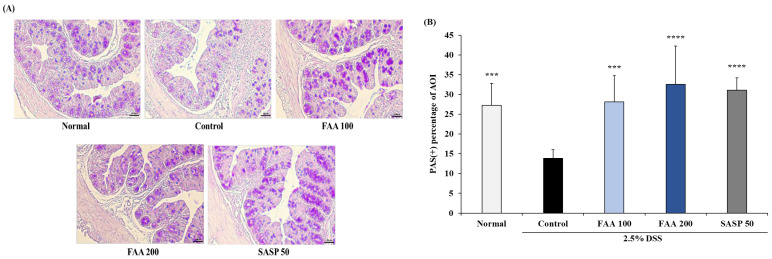
Effects of fermented *A. argyi* H. (FAA) on histological analysis for goblet cells in the DSS-induced colitis model. (**A**) The colon section was stained with PAS. Original magnification: 10× (bar = 100 μm). (**B**) The AOI was analyzed, and percentage of positive area was measured by ImageJ software. Values are mean ± SD (*n* = 9). *** *p* < 0.001, **** *p* < 0.001 vs. Control by one-way ANOVA and Tukey’s multiple comparison test. The mice were grouped and treated as described in Figure 4.

Goblet cells play an initiating role in protecting the intestinal barrier by secreting mucus, which strengthens the intestinal barrier. The number of goblet cells was confirmed through PAS staining through AOI (Figure 8). The number of goblet cells in the DSS-treated group was lower than that in the control groups. However, FAA 100 and FAA 200 significantly increased the number of goblet cells compared to the control group. These results demonstrated that FAA contributes to normal mucus secretion and intestinal barrier protection.

### 3.7. Effects of FAA on the Gut Microbiota Composition in the DSS-Induced Colitis Model

**Figure 9:**
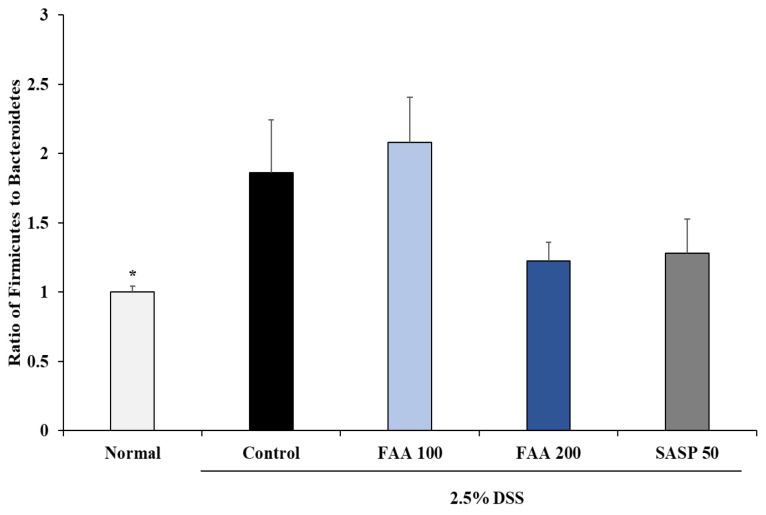
Effects of fermented *A. argyi* H. (FAA) on the gut microbiota composition in the DSS-induced colitis model. Values are mean ± SD (*n* = 9). * *p* < 0.05 vs. Control by one-way ANOVA and Tukey’s multiple comparison test. The mice were grouped and treated as described in Figure 4.

The Firmicutes/Bacteroidetes ratio influences the health of the gut; therefore, it was higher in the inflammatory models than in the normal group. The regulation of the gut microbiota imbalance was evaluated by the Firmicutes/Bacteroidetes ratio (Figure 9). There was an increase in the ratio in the DSS–only group compared with that in the normal group. Meanwhile, a decreasing tendency in the Firmicutes/Bacteroidetes ratio was exhibited in both the FAA 200 and SASP 50 groups, but the differences were nonsignificant compared with the control group.

### 3.8. Effects of FAA on Inflammation in the DSS-Induced Colitis Model

**Figure 10:**
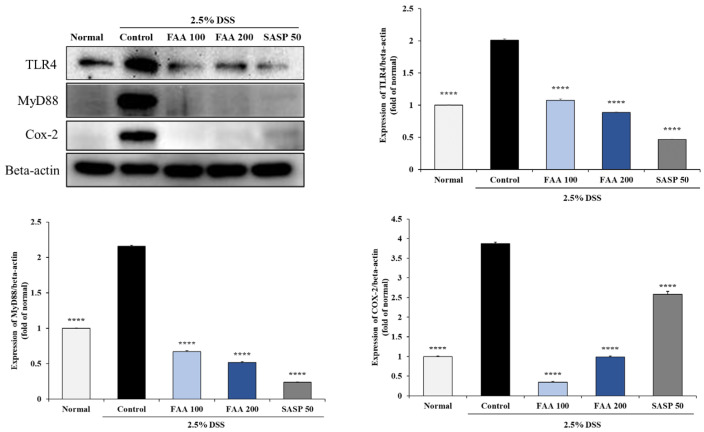
Effects of fermented *A. argyi* H. (FAA) on inflammation in the DSS-induced colitis model. Values are mean ± SD (*n* = 9). **** *p* < 0.0001 vs. Control by one-way ANOVA and Tukey’s multiple comparison test. The mice were grouped and treated as described in Figure 4.

The effects of FAA on the protein expression of inflammation-related factors in the colon were investigated (Figure 10). The protein expression of TLR4 at the cell membrane was significantly increased in the control group compared to that in the normal group, indicating that DSS treatment induces inflammatory responses in the colon. Moreover, in the MyD88-related pathway, the expression of MyD88 was upregulated in the control group compared with that in the normal group. However, FAA significantly inhibited the expression of TLR4 and MyD88 compared with the control group. Among the inflammatory mediators, the activities of COX-2 increased in the DSS-only group compared to those in the normal group, whereas FAA significantly downregulated COX-2 expression compared to the control group. These results suggest that FAA may attenuate inflammatory responses in the colon induced by colitis.

## 4. Discussion

This study confirmed the anti-inflammatory effect of FAA as a preventive agent against colitis, which is a type of IBD. In previous studies, the physicochemical properties, immune enhancement, and antioxidant activities of FAA were determined, but the anti-inflammatory effect through the prevention of colitis has not been proven to date.

Macrophage homeostasis is disrupted in colitis, which increases the expression of various inflammatory factors [35]. To test this hypothesis, it was measured by the production of inflammatory factors in LPS-induced RAW 264.7 cells in vitro. LPS treatment increases the expression of inducible nitric oxide (iNOS) in cells. The activation of iNOS oxidizes L-arginine within the cells and increases NO production [36]. Increased NO production promotes inflammation, and in severe cases, causes cell damage [37]. LPS-induced inflammation has a critical role in regulating immune responses and alleviating cell damage; thus, an appropriate balance of maintenance is necessary [38]. To verify the effect of regulating immune function by alleviating the inflammation generated by LPS without causing cell damage, inflammation was induced by treatment with LPS at a concentration of 100 ng/mL. Therefore, inflammation was induced by increasing the amount of NO production, and it was confirmed that NO production was significantly reduced by treatment with FAA at concentrations of 10, 50, 100, and 200 μg/mL, with no cytotoxicity. Additionally, LPS activates TLR4, a cell membrane receptor, thereby inducing an inflammatory signaling pathway within cells [39]. In this process, the production of various pro-inflammatory cytokines (TNF-α, IL-1β, IL-6, etc.) increases, and anti-inflammatory cytokines (IL-10, etc.) decrease [40]. In this study, FAA inhibited the production of pro-inflammatory cytokines such as IL-1β, and IL-6, thereby demonstrating its anti-inflammatory effect.

DSS is a chemical substance mainly used to study colon inflammation and disease [41]. Manifestations of colitis can be confirmed by clinical, histological, and pathological changes [42]. Generally, when colitis occurs, colon length decreases. Inflammation within the colon causes it to become shorter than normal, which directly confirms the manifestations of colitis [43]. Although colon length was similar to that of the group treated with DSS only, it was shorter in the group treated with FAA than in the normal group. These results indicated that DSS induced colitis. This was confirmed as the cause.

DSS treatment for 7–10 days is essential to induce colitis. Additionally, to determine the inflammatory state during the DSS treatment period, indicators (such as rectal bleeding, stool status, and degree of weight loss) must be measured [44]. The common symptoms of colitis include bloody stools, diarrhea, weight loss, and abdominal pain [45]. A DAI score between 0 and 3 indicates the onset of these symptoms, and changes in the inflammatory state can be confirmed using the DAI score [46]. The higher the DAI score, the higher the severity of colitis, and the progression of colitis over time can be identified [47]. Clinical symptoms such as bloody stools, diarrhea, and severe weight loss are caused by the induction of colitis [48]. Conversely, FAA treatment alleviated the clinical symptoms of colitis, confirming its anti-inflammatory effects.

The MLN plays a critical role in intestinal immune surveillance and regulation. It acts as a hub for immune cells and regulates the immune responses to antigens in the gut [49]. Subsequently, antigens are sampled and processed in the gut and attacked by T and B cells to indicate an immune response or induce resistance in gut microorganisms [50]. When intestinal inflammation occurs, an imbalance in intestinal immune cells, such as MLN cells, occurs through the production of pro-inflammatory cytokines [51]. In particular, colitis causes an imbalance between regulatory and effector T cells. When acute colitis is induced, the expression of TNF-α and IFN-γ increases the factors related to Th1 and Th17, and inflammatory responses are generated [52]. Therefore, the changes in the inflammatory state were confirmed by measuring the production of TNF-α and IFN-γ among the cytokines associated with Th1 cells, effector T cells, and MLN cells [53]. Consequently, DSS treatment caused an imbalance of the immune cells and colitis inflammation through increased production of TNF-α and IFN-γ, but FAA inhibits the production of these cytokines, regulating intestinal immune cell function and alleviating inflammation.

Normal colonic tissue is covered with mucosal cells and maintains an expanded crypt shape [54]. However, when inflammation occurs in the colon, histopathological indicators such as edema, leukocyte infiltration, crypt damage, and loss of goblet cells are observed [55]. When colitis occurs, edema occurs between the intestinal mucosa and submucosal tissue, and the infiltration of inflammatory cells into the colon increases [56]. Moreover, ulcers and blood clots occur, and the crypts collapse, causing damage to the intestinal epithelium [57]. However, after treatment with FAA, edema and infiltration of inflammatory cells were reduced, the state of the crypt was maintained at a level similar to that of the normal state, and histological damage improved.

Goblet cells are present in the inner membrane of the colon and produce mucus that protects and shares the inner membrane [58]. When colitis occurs, the number and function of goblet cells in the inner membrane of the colon may be impaired. Therefore, goblet cells stop producing mucus, which can damage the inner membrane of the colon [59]. However, FAA treatment increased the number of goblet cells to a level similar to that of the normal group, which increased the production of mucus and contributed to the protection of the intestinal barrier.

When colitis occurs, a gut microbiota imbalance occurs due to an increase in harmful microorganisms, causing the immune system to mistakenly attack the intestinal lining and worsen inflammation [60]. Recovery from colitis is confirmed through evaluation of the harmful and beneficial bacteria that control the composition and balance of the gut microorganisms, namely Firmicutes and Bacteroidetes, respectively [61]. Firmicutes are Gram-positive bacteria that extract and absorb energy from food; however, as metabolic diseases occur, the abundance of Firmicutes increases [62]. Conversely, Bacteroidetes are Gram-negative bacteria that play a critical role in the balance of gut microorganisms, breaking down fiber and other complex carbohydrates, and helping absorb nutrients [63]. When colitis is induced, the proportion of Firmicutes, which is known to be relatively harmful, increases, and the proportion of Bacteroidetes, which is known to be relatively beneficial, decreases, resulting in an imbalance of intestinal microorganisms [64]. Therefore, by treatment with FAA, the control and balance of gut microorganisms were confirmed by reducing the relative ratio of Firmicutes/Bacteroidetes, and its tendency to inhibit intestinal inflammation was determined, but more-detailed investigations of the microbiota species are required.

Inflammatory material, such as DSS, destroys the intestinal barrier to produce endotoxins (i.e., LPS), which stimulates the LPS receptor TLR4. Stimulated TLR4 initiates the MyD88 signaling pathway, which is attracted to the intracellular region [65], resulting in the production of inflammatory mediators such as COX-2 [66,67]. Consequently, the expression of COX-2 induces the overexpression of iNOS and prostaglandins, triggering an inflammatory response via the production of pro-inflammatory cytokines [68]. Evidence demonstrated that TLR4 is highly expressed in DSS-induced colitis mice. In this study, DSS destroyed the intestinal barrier in mice, resulting in the high expression of TLR4, MyD88, and COX-2. However, treatment with FAA downregulated the expression of these inflammatory proteins. Similarly, recent findings have shown that the TLR4/MyD88 signaling pathway is strongly linked with the inflammatory response in the colitis mouse model and fulfills a crucial role in the pathogenesis of colitis. Therefore, inhibiting the TLR4/MyD88/COX-2 signaling pathway in present study was shown to be a valuable approach for the prevention and improvement of colitis.

## 5. Conclusions

This study aimed to investigate the inhibitory effects of FAA on inflammation. In vitro, the anti-inflammatory effect was demonstrated by reducing the production of NO and pro-inflammatory cytokines (TNF-α, IL-6, and IL-1β) in LPS-induced RAW 264.7 cells. Moreover, in vivo, the anti-inflammatory effect was confirmed by decreasing the intestinal length, DAI score, the level of inflammatory cytokines, infiltration of inflammatory cells, and improving the crypt damage in the DSS-induced colitis model. Downregulated expression of inflammatory proteins (TLR4, MyD88, and COX-2) in colon tissues also indicated the anti-inflammatory effect of FAA. Taken together, these results suggests that FAA can be used as a functional material for gut health to alleviate intestinal inflammation. However, further study is needed to investigate comprehensive mechanisms of FAA and explore its potential effect on the intestinal mucosa (i.e., tight junctions) at the molecular level.

## Figures and Tables

**Table 1:** The specific primers of 16S rRNA gene-targeted group.

Target Group	Sequences		Annealing Temperature (°C)
	Forward	Reverse	
Uni (F341/R518)	CCTACGGGAGGCAGCAGT	ATTACCGCGGCTGCTGG	59
Firmicutes (Phylum)	TGAAACTYAAAGGAATTGACG	ACCATGCACCACCTGTC	60
Bacteroidetes (Phylum)	GGARCATGTGGTTTAATTCGATGAT	AGCTGACGACAACCATGCAG	60

**Table 2:** DAI scores of colitis by means of clinical parameters during DSS treatment.

Score	Rectal Bleeding	Stool Consistency	Weight Loss (% of Initial wt)
0	Normal	Normal pellets	<1%
1	Slightly bloody	Slightly loose feces	1–4.99%
2	Bloody	Loose feces	5–10%
3	Bloody in whole colon	Watery diarrhea	>10%

Percentage of body weight loss = (initial weight − current weight)/(initial weight) × 100. DAI = (rectal bleeding + stool consistency + weight loss)/3.

**Table 3:** Scoring of colitis through the colon sections by histological analysis.

Score	Inflammation Severity	Inflammation Extent	Crypt Damage
0	None	None	Damage to the basal third of the crypt
1	Slight	Mucosal	Damage to the basal two-thirds of the crypt
2	Moderate	Submucosal	Only surface epithelium intact
3	Severe	Transmural	Loss of entire crypt and epithelium

## Data Availability

The original contributions presented in the study are included in the article, further inquiries can be directed to the corresponding author.
